# Fatal Case of Cerebral Affection, with Symptoms Resembling Hysteria, in Which Yellow Tubercles Were Discovered in the Brain and Cerebellum

**Published:** 1826-07

**Authors:** 

**Affiliations:** the Middlesex Hospital.


*Fatal Case of Cerebral Affection, with Symptoms resembling Hysteria, in which yellow Tubercles were discovered in the Brain and Cerebellum.* Treated by Dr. HAWKINS, at the MIDDLESEX HOSPITAL.

JOHN YOUDEN, aged twenty-three; admitted into the Middlesex Hospital, April 11th, 1826.

This man, during the last eight or nine weeks, has been subject to frequent fits, which, from his own account, together with the description afforded us by his friends, appear to have been epileptic. He complains of a severe pain in his hips and loins; he is affected with dysuria, and hemorrhoids of a very painful nature. These latter are most probably the cause of the dysuria and pain in the hips. He has partially lost the power of moving the lower extremities, but still they cannot be said to be paralytic, since he can move them in any direction, though with difficulty. The bowels are confined, and the tongue furred.

May 1st.—He is unable to retain his urine, it is found dribbling from him. He has had no fit since he came to the hospital.

8th.—He has had a fit to-day, the first since his admission. The power of articulation is evidently affected; his vision is imperfect; the control over the muscles of the left arm is in some degree impaired. Pulse 120, small and thready. The involuntary discharge of the contents of the bladder no longer continues, and the urine is retained: it is, therefore, necessary to draw it off.

21st.—The fits closely resemble those of hysteria in women; he is not entirely deprived of his senses by them: whilst they continue, the pulse is frequently found to rise to 150 in the minute. He complains of giddiness when the head is raised. The fits usually commence with a shivering, similar to that of an intermittent fever. He complains of cold, whilst the skin is moist and warm.

The operation of drawing off his urine first led to the observation of the extraordinary quantity of that fluid secreted: probably it amounts to not less than three quarts a-day.

Three or four days previous to his decease, the fæces were passed involuntarily; and he remained in a comatose state until his death, on the 29th.

It is unnecessary to detail at full length the medical treatment, which in a case of this nature must necessarily have been chiefly palliative. The oleum terebinthinæ was at first tried, as recommended by Dr. Prichard, and supposed by him to be almost a specific in certain cases of epilepsy. But, though it failed to produce any effect on the nervous symptoms, it was of some use in mitigating the dysuria; and when it was afterward laid aside, the patient himself requested that it might be resumed, on account of the relief which he had experienced whilst taking it. The infusions of uva ursi and of buchu failed to produce any benefit. The common medicines and applications for hemorrhoids produced no effect; but an opiate suppository gave great temporary relief, and succeeded in procuring sleep. Leeches and blisters were tried, but without avail; and a variety of antispasmodic medicines, with as little effect. Mercury was at first freely given as a purgative, and afterwards with the view to affect the system, but without benefit in either way.

This brief outline of the case is intended chiefly to connect the symptoms, and the morbid appearances which were presented on dissection.

*Sectio cadaveris.*—The brain was exposed, and a slight opacity of the arachnoid membrane was the first deviation from healthy structure observable. The substance of the brain was unusually firm in its texture, and the ventricles were found to contain about an ounce of serum. On examining very minutely the intimate structure, small tubercles, of a yellow colour, as described by M. Wenzel, were discovered, occupying both the cerebrum* and cerebellum: those found in the cerebrum were situated, for the most part, near its base; and, near the cineritious convoluted surface, they were about four or five in number. The tubercles, however, were most extensive in the cerebellum: here they

* We are not aware that Wenzel has described these tubercles as situated in the cerebrum.

were found deeply imbedded in its substance, and occasionally connected by filaments of a similarly organised matter.* On making a section of several of these, they were found closely to resemble the yellow scrofulous tubercle of the lungs. Some, being denser than others, were cut with more difficulty: these were invariably the smallest;—others were larger, and much softer in their texture, and as it were more fully developed, agreeing in this respect with the same formations in the other structures.†

A small hydatid was found in the liver, projecting slightly on the anterior surface of the larger lobe; and a small collection of pus was found on the sternum, immediately under the integument.

The physician observed, that diseases of the brain, by their universal influence, were very apt to produce changes of structure in various organs of the body. As it requires very minute examination to discover these tubercles, it is not improbable that this, as well as other diseased appearances in epilepsy, are often overlooked; and that Lobstein is not far wrong when he states that there *ought* always to be found some diseased appearances within the cranium: of course, by this, implying that less inveterate cases, not proving fatal, may arise from other causes.

There were several curious circumstances attending this case. We believe that no doubt now exists of men being occasionally the subjects of a disease, exhibiting precisely the same symptoms as hysteria in women; nor is there more doubt of the latter disease being very frequently attended by symptoms not distinguishable from those of epilepsy: the disorders, in fact, are shaded into one another by insensible gradations. Both probably arise, as Dr. Parr observes, from the same cause, an excessive degree of nervous excitement or irritation. The correctness of the above observation was in this case very manifest: he had fits of a truly hysterical character; the urine was pale, and secreted in great quantity; and his pulse was exceedingly rapid.‡

* It may be remarked, that there never were in this case the irregular and convulsive movements which have been attributed by some foreign physiologists to injuries of the cerebellum.

† Dr. Baillie has observed, that it is not very unusual to see a white substance formed in the brain, of an uniform smooth texture, and possessing a considerable degree of hardness, which he has ascertained to be scrofulous in its nature, having had an opportunity of seeing it converted into a scrofulous pus. But, with respect to the origin and nature of the yellow tubercles in the present case, it must be admitted that there were no traces of scrofulous formations or degeneration in other parts of the body. The lungs were remarkably healthy in their structure.

‡ We are sorry we had no opportunity of counting his pulse during sleep; for this is the best mark of distinction between hysteria and the various inflammatory diseases which it mimics. During sleep, it generally falls to near its natural standard in hysterical affections.

